# Supplementary material for: Polypharmacy Management in Chronic Conditions: A Systematic Literature Review of Italian Interventions
Source: J Clin Med. 2024 Jun 17;13(12):3529. doi: 10.3390/jcm13123529 (PMC11204469; doi:10.3390/jcm13123529)
Supplement: Supplementary file 1 [file jcm-13-03529-s001.zip › jcm-3028549-supplementary.pdf]

Supplementary Material

Supplementary Table S1. Search strategy.

Database	Query performed
**PubMed**	("polypharmacy"[Title/Abstract] OR "polytherapy"[Title/Abstract] OR "polypragmasy"[Title/Abstract] OR "polymedication"[Title/Abstract] OR "multiple medication"[Title/Abstract] OR "multiple medicine"[Title/Abstract] OR "multiple drug"[Title/Abstract] OR (("medic"[All Fields] OR "medical"[All Fields] OR "medicalization"[MeSH Terms] OR "medicalization"[All Fields] OR "medicalizations"[All Fields] OR "medicalize"[All Fields] OR "medicalized"[All Fields] OR "medicalizes"[All Fields] OR "medicalizing"[All Fields] OR "medically"[All Fields] OR "medicals"[All Fields] OR "medicated"[All Fields] OR "medication s"[All Fields] OR "medics"[All Fields] OR "pharmaceutical preparations"[MeSH Terms] OR ("pharmaceutical"[All Fields] AND "preparations"[All Fields]) OR "pharmaceutical preparations"[All Fields] OR "medication"[All Fields] OR "medications"[All Fields]) AND "overload"[Title/Abstract]) OR "multiple medication use"[Title/Abstract] OR (("excess"[All Fields] OR "excesses"[All Fields] OR "excessive"[All Fields] OR "excessively"[All Fields]) AND "drug therapy"[Title/Abstract]) OR "medication polytherapy"[Title/Abstract] OR "medication complexity"[Title/Abstract] OR "drug regimen complexity"[Title/Abstract] OR "medication burden"[Title/Abstract] OR (("medic"[All Fields] OR "medical"[All Fields] OR "medicalization"[MeSH Terms] OR "medicalization"[All Fields] OR "medicalizations"[All Fields] OR "medicalize"[All Fields] OR "medicalized"[All Fields] OR "medicalizes"[All Fields] OR "medicalizing"[All Fields] OR "medically"[All Fields] OR "medicals"[All Fields] OR "medicated"[All Fields] OR "medication s"[All Fields] OR "medics"[All Fields] OR "pharmaceutical preparations"[MeSH Terms] OR ("pharmaceutical"[All Fields] AND "preparations"[All Fields]) OR "pharmaceutical preparations"[All Fields] OR "medication"[All Fields] OR "medications"[All Fields]) AND "cascade"[Title/Abstract]) OR "multitherapy"[Title/Abstract] OR "polypharmacotherapy"[Title/Abstract] OR (("polymedicated"[All Fields] OR "polypharmacy"[MeSH Terms] OR "polypharmacy"[All Fields] OR "polymedication"[All Fields]) AND "therapy"[Title/Abstract]) OR "multi drug therapy"[Title/Abstract] OR "multiple drug treatment"[Title/Abstract] OR "polydrug therapy"[Title/Abstract]) AND ("manage"[All Fields] OR "managed"[All Fields] OR "management s"[All Fields] OR "managements"[All Fields] OR "manager"[All Fields] OR "manager s"[All Fields] OR "managers"[All Fields] OR "manages"[All Fields] OR "managing"[All Fields] OR "managment"[All Fields] OR "organization and administration"[MeSH Terms] OR ("organization"[All Fields] AND "administration"[All Fields]) OR "organization and administration"[All Fields] OR "management"[All Fields] OR "disease management"[MeSH Terms] OR ("disease"[All Fields] AND "management"[All Fields]) OR "disease management"[All Fields] OR ("governability"[All Fields] OR "governable"[All Fields] OR "governance"[All Fields] OR "governances"[All Fields] OR "government"[MeSH Terms] OR "government"[All Fields] OR "governments"[All Fields] OR "government s"[All Fields]) OR

("administrable"[All Fields] OR "administrate"[All Fields] OR "administrated"[All Fields] OR "administrating"[All Fields] OR "administrations"[All Fields] OR "administred"[All Fields] OR "administrered"[All Fields] OR "organization and administration"[MeSH Terms] OR ("organization"[All Fields] AND "administration"[All Fields]) OR "organization and administration"[All Fields] OR "administration"[All Fields]) OR ("governability"[All Fields] OR "governable"[All Fields] OR "governance"[All Fields] OR "governances"[All Fields] OR "government"[MeSH Terms] OR "government"[All Fields] OR "governments"[All Fields] OR "government s"[All Fields]) OR ("handle"[All Fields] OR "handled"[All Fields] OR "handles"[All Fields] OR "handling"[All Fields] OR "handlings"[All Fields]) OR ("operability"[All Fields] OR "operable"[All Fields] OR "operate"[All Fields] OR "operated"[All Fields] OR "operates"[All Fields] OR "operating"[All Fields] OR "operation s"[All Fields] OR "operational"[All Fields] OR "operative"[All Fields] OR "operatively"[All Fields] OR "operatives"[All Fields] OR "operator"[All Fields] OR "operator s"[All Fields] OR "operators"[All Fields] OR "surgery"[MeSH Subheading] OR "surgery"[All Fields] OR "operations"[All Fields] OR "surgical procedures, operative"[MeSH Terms] OR ("surgical"[All Fields] AND "procedures"[All Fields] AND "operative"[All Fields]) OR "operative surgical procedures"[All Fields] OR "operation"[All Fields]) OR ("controling"[All Fields] OR "controllability"[All Fields] OR "controllable"[All Fields] OR "controllably"[All Fields] OR "controller"[All Fields] OR "controller s"[All Fields] OR "controllers"[All Fields] OR "controlling"[All Fields] OR "controls"[All Fields] OR "prevention and control"[MeSH Subheading] OR ("prevention"[All Fields] AND "control"[All Fields]) OR "prevention and control"[All Fields] OR "control"[All Fields] OR "control groups"[MeSH Terms] OR ("control"[All Fields] AND "groups"[All Fields]) OR "control groups"[All Fields]) OR ("organization and administration"[MeSH Terms] OR ("organization"[All Fields] AND "administration"[All Fields]) OR "organization and administration"[All Fields] OR "supervision"[All Fields] OR "supervise"[All Fields] OR "supervised"[All Fields] OR "supervises"[All Fields] OR "supervising"[All Fields] OR "supervisions"[All Fields]) OR ("command"[All Fields] OR "command s"[All Fields] OR "commandant"[All Fields] OR "commanded"[All Fields] OR "commander"[All Fields] OR "commander s"[All Fields] OR "commanders"[All Fields] OR "commanding"[All Fields] OR "commands"[All Fields]) OR ("operability"[All Fields] OR "operable"[All Fields] OR "operate"[All Fields] OR "operated"[All Fields] OR "operates"[All Fields] OR "operating"[All Fields] OR "operation s"[All Fields] OR "operational"[All Fields] OR "operative"[All Fields] OR "operatively"[All Fields] OR "operatives"[All Fields] OR "operator"[All Fields] OR "operator s"[All Fields] OR "operators"[All Fields] OR "surgery"[MeSH Subheading] OR "surgery"[All Fields] OR "operations"[All Fields] OR "surgical procedures, operative"[MeSH Terms] OR ("surgical"[All Fields] AND "procedures"[All Fields] AND "operative"[All Fields]) OR "operative surgical procedures"[All Fields] OR "operation"[All Fields]) OR ("running"[MeSH Terms] OR "running"[All Fields] OR "runnings"[All Fields])) AND ("adult"[Title/Abstract] OR "adult patient"[Title/Abstract] OR "adult subject"[Title/Abstract] OR "adult population"[Title/Abstract] OR "elderly"[Title/Abstract] OR "old"[Title/Abstract] OR "aged"[Title/Abstract] OR "senior"[Title/Abstract] OR

	"veteran"[Title/Abstract] OR "older adult"[Title/Abstract] OR "older geriatric"[Title/Abstract] OR "geriatric patient"[Title/Abstract] OR "aged individual"[Title/Abstract] OR "aged subject"[Title/Abstract] OR (("elder s"[All Fields] OR "elders"[All Fields] OR "sambucus"[MeSH Terms] OR "sambucus"[All Fields] OR "elder"[All Fields]) AND "subject"[Title/Abstract]) OR "elder population"[Title/Abstract] OR "aging population"[Title/Abstract] OR (("aging"[MeSH Terms] OR "aging"[All Fields] OR "ageing"[All Fields]) AND "senior citizen"[Title/Abstract])) AND ("intervention"[Title/Abstract] OR "educational"[Title/Abstract] OR "educative"[Title/Abstract] OR "instructive"[Title/Abstract] OR "instructional"[Title/Abstract] OR "instructional"[Title/Abstract] OR "strategy"[Title/Abstract]) AND ("cost"[Title/Abstract] OR "healthcare cost"[Title/Abstract] OR "avoidable cost"[Title/Abstract] OR "burden"[Title/Abstract] OR "expense"[Title/Abstract] OR "budget"[Title/Abstract] OR "spending"[Title/Abstract] OR "outlay"[Title/Abstract] OR "overheads"[Title/Abstract] OR "expenditure"[Title/Abstract]) AND ("italy"[MeSH Terms] OR "italy"[All Fields] OR "italy s"[All Fields] OR ("italian people"[Supplementary Concept] OR "italian people"[All Fields] OR "italians"[All Fields] OR "italian"[All Fields]))
**Embase**	(polypharmacy:ab,ti OR polytherapy:ab,ti OR polypragmasy:ab,ti OR polymedication:ab,ti OR 'multiple medication':ab,ti OR 'multiple medicine':ab,ti OR 'multiple drug':ab,ti OR 'medication overload':ab,ti OR 'multiple medication use':ab,ti OR 'excessive drug therapy':ab,ti OR 'medication polytherapy':ab,ti OR 'medication complexity':ab,ti OR 'drug regimen complexity':ab,ti OR 'medication burden':ab,ti OR 'medication cascade':ab,ti OR multitherapy:ab,ti OR polypharmacotherapy:ab,ti OR 'polymedication therapy':ab,ti OR 'multi-drug therapy':ab,ti OR 'multiple drug treatment':ab,ti OR 'polydrug therapy':ab,ti) AND (management OR government OR administration OR governance OR handling OR control OR supervision OR command OR operation OR running) AND (adult:ab,ti OR 'adult patient':ab,ti OR 'adult subject':ab,ti OR 'adult population':ab,ti OR elderly:ab,ti OR old:ab,ti OR aged:ab,ti OR senior:ab,ti OR veteran:ab,ti OR 'older adult':ab,ti OR 'older geriatric':ab,ti OR 'geriatric patient':ab,ti OR 'aged individual':ab,ti OR 'aged subject':ab,ti OR 'elder subject':ab,ti OR 'elder population':ab,ti OR 'aging population':ab,ti OR 'aging senior citizen':ab,ti) AND (intervention:ab,ti OR educational:ab,ti OR educative:ab,ti OR instructive:ab,ti OR instructional:ab,ti OR strategy:ab,ti) AND (cost:ab,ti OR 'healthcare cost':ab,ti OR 'avoidable cost':ab,ti OR burden:ab,ti OR expense:ab,ti OR budget:ab,ti OR spending:ab,ti OR outlay:ab,ti OR overheads:ab,ti OR expenditure:ab,ti) AND (italy OR italian)
**ProQuest**	summary(polypharmacy OR polytherapy OR polypragmasy OR polymedication OR multiple medication OR multiple medicine OR multiple drug OR Medication overload OR Multiple medication use OR Excessive drug therapy OR medication polytherapy OR medication complexity OR drug regimen complexity OR Medication burden OR Medication cascade OR Multitherapy OR polypharmacotherapy OR polymedication therapy OR Multi-drug therapy OR Multiple drug treatment OR Polydrug therapy) AND (management OR government OR administration OR governance OR handling OR operation OR control OR supervision OR command OR operation OR running) AND summary(Adult OR adult patient OR adult subject OR adult population OR elderly OR old OR aged OR senior OR veteran OR Older adult OR Older Geriatric OR Geriatric patient OR

	Aged individual OR Aged subject OR Elder subject OR Elder population OR Aging population OR Aging Senior citizen) AND summary(intervention OR educational OR educative OR instructive OR instructional OR strategy) AND summary(cost OR healthcare cost OR Avoidable Cost OR burden OR expense OR budget OR spending OR outlay OR overheads OR expenditure) AND (italy OR italian) AND la.exact("English")
**Web of Science**	(((((AB=(polypharmacy OR polytherapy OR polypragmasy OR polymedication OR multiple medication OR multiple medicine OR multiple drug OR Medication overload OR Multiple medication use OR Excessive drug therapy OR Medication polytherapy OR Medication complexity OR Drug regimen complexity OR Medication burden OR Medication cascade OR Multitherapy OR Polypharmacotherapy OR polymedication therapy OR Multi-drug therapy OR Multiple drug treatment OR Polydrug therapy)) AND ALL=(management OR government OR administration OR governance OR handling OR operation OR control OR supervision OR command OR operation OR running)) AND AB=(Adult OR adult patient OR adult subject OR adult population OR elderly OR old OR aged OR senior OR veteran OR Older adult OR Older Geriatric OR Geriatric patient OR Aged individual OR Aged subject OR Elder subject OR Elder population OR Aging population OR Aging Senior citizen)) AND AB=(intervention OR educational OR educative OR instructive OR instructional OR strategy)) AND AB=(cost OR healthcare cost OR Avoidable Cost OR burden OR expense OR budget OR spending OR outlay OR overheads OR expenditure)) AND ALL=(Italy or italian)

Supplementary Table S2. Number of records obtained by search strategy.

Query	Keywords	Number of records			
		PubMed	Embase	ProQuest	Web of Science
#1	Polypharmacy OR polytherapy OR polypragmasy OR polymedication OR multiple medication OR multiple medicine OR multiple drug OR medication overload OR multiple medication use OR excessive drug therapy OR medication polytherapy OR medication complexity OR drug regimen complexity OR medication burden OR medication cascade OR multitherapy OR polypharmacotherapy OR polymedication therapy OR multi-drug therapy OR multiple drug treatment OR polydrug therapy	67,201	31,627	39,525	195,073
#2	Management OR government OR administration OR governance OR handling OR operation OR control OR supervision OR command OR operation OR running	14,940,568	12,128,519	16,728,829	17,121,370
#3	Adult OR adult patient OR adult subject OR adult population OR elderly OR old OR aged OR senior OR veteran OR older adult OR older geriatric OR geriatric patient OR aged individual OR aged subject OR elder subject OR elder population OR aging population OR aging senior citizen	3,023,887	3,128,490	1,038,471	4,537,409
#4	Intervention OR educational OR educative OR instructive OR instructional OR strategy	1,725,134	2,226,393	6,273,649	4,087,296
#5	Cost OR healthcare cost OR avoidable cost OR burden OR expense OR budget OR spending OR outlay OR overheads OR expenditure	964,083	1,291,548	803,334	2,755,175
#6	Italy OR italian	1,360,175	2,291,824	2,846,791	3,117,838
#7	#1 AND #2 AND #3 AND #4 AND #5 AND #6	8	6	63	76

Supplementary Table S3. STROBE Analysis of observational studies.

Reference	SCORE Item Number																						COR score
	1	2	3	4	5	6	7	8	9	10	11	12	13	14	15	16	17	18	19	20	21	22	
**Radaelli F, et al. 2021**	✓	✓	✓	✓	✓	✓	✓	✓		✓	✓	✓	✓	✓	✓	✓		✓	✓	✓	✓		High 86%
**Faverio P, et al. 2020**	✓	✓	✓		✓	✓	✓	✓	✓	✓	✓	✓	✓	✓	✓	✓	✓	✓	✓	✓	✓	✓	High 90%
**Atella V, et al. 2019**	✓	✓	✓		✓	✓	✓	✓	✓	✓	✓	✓	✓	✓	✓			✓	✓	✓	✓		High 82%
**Focà E, et al. 2019**	✓	✓	✓	✓	✓	✓	✓	✓		✓	✓	✓	✓	✓	✓		✓	✓	✓	✓	✓	✓	High 90%
**Grion AM, et al. 2016**	✓	✓	✓	✓	✓	✓	✓	✓		✓	✓	✓	✓	✓	✓	✓		✓	✓	✓	✓		High 86%
**Dal Negro RW, et al. 2016**	✓	✓	✓	✓	✓	✓	✓	✓		✓	✓	✓	✓	✓	✓			✓	✓	✓	✓		High 81%
**Foo J, et al. 2016**	✓	✓	✓	✓	✓	✓	✓	✓		✓	✓	✓	✓	✓	✓		✓	✓	✓	✓	✓	✓	High 93%

Check symbols indicate the presence of these items in the chosen studies. Blank spaces indicate the absence of the item. The evidence column indicates the number of STROBE items present in the article relative to the total number of STROBE items. The quality of studies was measured according to the Completeness of Reporting (COR) score: “low” (COR: 0–49%), “moderate” (COR: 50–74%) and “high” if ≥75% of items were met.

Supplementary Table S4. CASP Analysis of experimental studies.

Reference	CASP Section				COR score
	A	B	C	D	
**Ghibelli S, et al. 2013**	✓		✓	✓	High 75%
**Onder G, et al. 2008**	✓		✓	✓	High 75%

Check symbols indicate the presence of these items in the chosen studies. Blank spaces indicate the absence of the item. The evidence column indicates the number of STROBE items present in the article relative to the total number of STROBE items. The quality of studies was measured according to the Completeness of Reporting (COR) score: “low” (COR: 0–49%), “moderate” (COR: 50–74%) and “high” if ≥75% of items were met.

Supplementary Table S5. Characteristics and main outcomes of included observational studies.

Authors	Year	Number of patients	Sample age	Levels of Polypharmacy	Levels of Comorbidity
**Radaelli F, et al.**	2021	1.198	Median age (IQR): 78 years (67-84)	Prevalence (n)<br>-Single antiplatelet agent (ASA or clopidogrel or ticlopidine): 285 (23.8)<br>-Dual antiplatelet therapy: 42 (3.5)<br>-Direct oral anticoagulant: 143 (11.8)<br>-Vitamin K antagonist: 115 (9.6)	Charlson-Comorbidity Index (n %):<br>-0: 336 (28.0)<br>-1: 274 (22.9)<br>-2: 214 (17.9)<br>-≥3: 374 (31.2)<br><br>Comorbid illness (prevalence n %):<br>-Atrial fibrillation/ Venous thromboembolism: 333 (2.8)<br>-Coronary heart disease: 293 (24.4)<br>-Moderate to severe renal disease: 205 (17.1)
**Faverio P, et al.**	2020	7.322	Mean age (SD): 71.2 years (16.3)	Prevalence (n %):<br>-Antihypertensives: n=120,543 (59.2%)<br>-Drugs for obstructive airway Diseases: n=46,239 (22.7%)<br>-Inhaled steroids: n=35,584 (17.5%)<br>-Inhaled broncodilators β: n=29,012 (14.2%)	Multisource Comorbidity Score α: Low 139.7 (68.6%) High of 64.0 (31.4%)
**Atella V, et al.**	2019	1.035.984	Age groups:<br>-35–50 patients<br>-81+ patients	Average number of DDD prescribed (trend 2005-2014):<br>-35–50 age group: from 114 to 119.9<br>-81+ age group: from 774.9 to 1,178.1	\
**Focà E, et al.**	2019	210	Median age (IQR):<br>-Overall: 69.8 (67.1–73.9)<br>-F: 70.6 (67.3–74.8)<br>-M: 69.6 (67.1–73.8)	Prevalence (n %):<br>-Overall: 254 (20.5%)<br>-F: 42 (20%)<br>-M: 234 (22.8%))	Prevalence (%):<br>-cardiovascular disease 20.46% (F: 9.5%; M: 22.8%)<br>-bone disease 28.5% (F: 48.8%; M: 22.9%)
**Grion AM, et al.**	2016	Validation cohort: 449	Median age (IQR):<br>86 (81–90)	Prevalence (n %):<br>-Number of drugs at admission: 6 (4–8)	MPI (Grades MPI 1 vs. MPI 2–3): 0.69 (0.50–0.81)

				Variables independently associated with PIMs at admission (OR):<br>-Polytherapy (0–4 vs. C5 drugs): 3.01 (2.30–3.94)<br>-Drugs used in diabetes*: 0.76 (0.38–1.51)<br>-Antithrombotic agents*: 2.06 (1.17–3.61)<br>-Cardiac glycosides*: 1.49 (0.57–3.94)<br>-Antihypertensive agents*: 1.31 (0.66–2.61)	
**Dal Negro RW, et al.**	2016	817	Mean age (SD):<br>-Overall 49.06 (±0.64),<br>-Group A 43.42 (±0.78),<br>-Group B 57.46 (±1.16),<br>-Group C 60 (±1.17)	Mean (±SE, standard error): Concomitant therapies 50.65 (±4.34) €/patient/year	Prevalence (%):<br>-Overall: 69.16%<br>-Group A: 59.84%<br>-Group B: 81.68%<br>-Group C: 89.72%
**Foo J, et al.**	2016	Overall cohort: 4.343<br>Italian cohort: 302	Mean age (SD): 63.1 (12.8)	\	Prevalence (n):<br>-Comorbidities 0-1: n=71<br>-Comorbidities ≥2: n=28

Abbreviations: female population (F); interquartile (IQR); male population (M); multidimensional prognostic index (MPI); odds ratio (OR); Potentially inappropriate medications (PIMs); standard deviation (SD).

** Drugs used in diabetes (A10); Antithrombotic agents (B01); Cardiac glycosides (C01A); Antihypertensive agents (C02, C03A, C03B, C03C, C07, C08C, C09).*

Supplementary Table S6. Characteristics and main outcomes of included experimental studies.

Authors	Year	Number of patients	Sample age	Levels of Polypharmacy	Levels of Comorbidity
**Ghibelli S, et al.**	2013	Observational phase: 74<br>Experimental phase: 60	Mean age: 81.3	\	Comorbidity Index Score (mean): 4.6
**Onder G, et al.**	2008	Intervention group: 1.539<br>No intervention group: 2.468	Mean age: 82.3	\	\
